# Ischemic colitis in a child: a case report and narrative review

**DOI:** 10.3389/fped.2026.1791996

**Published:** 2026-04-29

**Authors:** Qin Zhong, Jie Zhao

**Affiliations:** 1 Pediatrics, Zibo Central Hospital, Zibo, Shandong, China; 2 School of Clinical Medicine, Shandong Second Medical University, Weifang, Shandong, China

**Keywords:** child, colonoscopy, diagnosis, hematochezia, ischemic colitis

## Abstract

**Introduction:**

Ischemic colitis is extremely rare in preschool-aged children and is still susceptible to misdiagnosis due to atypical symptoms. Misdiagnosis can have serious consequences, including delayed treatment and disease progression, so raising clinical awareness of this condition is critical. This report aims to contribute by providing a relevant case and a narrative overview of ischemic colitis in children.

**Case presentation:**

A 6-year-old boy presented with recurrent hematochezia for 6 days and mild abdominal pain. Laboratory tests showed elevated inflammatory markers. Colonoscopy revealed segmental mucosal congestion and erosions in the descending and sigmoid colon; pathology confirmed chronic active mucosal inflammation. After excluding infectious colitis and inflammatory bowel disease, ischemic colitis was diagnosed. Conservative management with bowel rest, intravenous fluids, and mucosal protective agents achieved complete symptom resolution without recurrence.

**Conclusion:**

In conjunction with the accompanying narrative overview, this case underscores that ischemic colitis, though rare, must be considered in the differential diagnosis of unexplained hematochezia in preschool children. The favorable outcome achieved through conservative management aligns with the broader literature synthesis, reinforcing that non—surgical intervention is both feasible and effective in this demographic. This review of the available evidence further highlights the critical need for heightened clinical suspicion to avoid delayed intervention.

## 1 Introduction

Ischemic colitis (IC) results from transient or sustained impairment of colonic blood perfusion, causing mucosal injury (1). Its key symptoms are acute abdominal pain and hematochezia. Predominantly affecting older adults, especially those with cardiovascular risk factors, IC has seen a four—fold increase since the late 20th century, likely due to better recognition, aging populations, and more diagnostic endoscopy use (2, 3). In contrast, pediatric IC is extremely rare, with few reports.

Clinically, IC often presents with mild or self—limited symptoms, leading to diagnostic uncertainty and misdiagnosis. It most commonly affects the left colon. Colonoscopy with histopathological biopsy is the diagnostic standard (4, 5). Most cases respond well to conservative management, but recurrence can occur over time (6, 7).

This report details a 6—year—old boy's IC case, aiming to enhance pediatric clinicians' diagnostic awareness for timely and accurate identification and appropriate care.

## 2 Case presentation

A 6-year-old boy was admitted to our hospital for evaluation of hematochezia persisting for 6 days. The hematochezia began insidiously without identifiable precipitant: bright-red blood, small in volume, odorless, occurring 3–8 times daily, and associated with abdominal discomfort during defecation. At illness onset, he developed a low-grade fever lasting one day and occasional nonproductive cough; there was no vomiting, dizziness, rash, or arthralgia/arthritis. His medical history included a prior episode of hematochezia attributed to lactose intolerance, which resolved with dietary lactose restriction and supportive care. Empiric antimicrobial and symptomatic therapy administered at an outside facility failed to improve symptoms; indeed, the frequency and volume of hematochezia increased one day prior to admission, prompting transfer for further diagnostic evaluation and management.

On admission, physical examination revealed: the patient was alert and well-appearing; pharyngeal mucosa showed mild congestion without exudate, and tonsils were non-enlarged; cardiopulmonary examination was unremarkable; the abdomen was soft, with mild tenderness localized to the periumbilical and lower abdominal regions; no guarding, rebound tenderness, or rigidity was present; bowel sounds were normoactive (approximately 5 per minute); and abdominal percussion demonstrated no shifting dullness.

**Figure 1:**
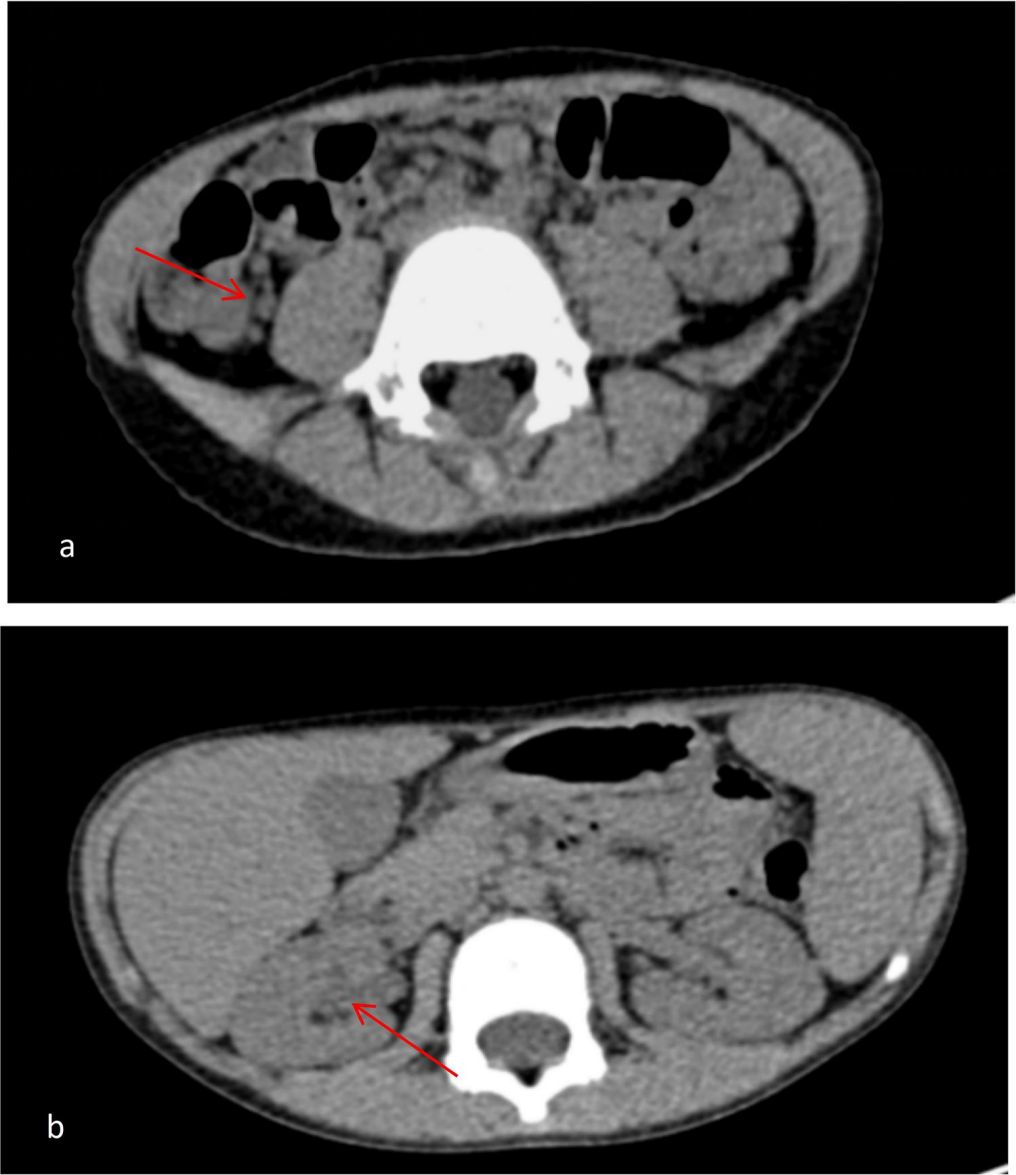
**(a)** Axial view at the level of the ileocecal region shows multiple small mesenteric lymph nodes (≤5 mm, red arrow). **(b)** Coronal view of the upper abdomen demonstrates mild dilation of the right renal pelvis (red arrow).

**Figure 2:**
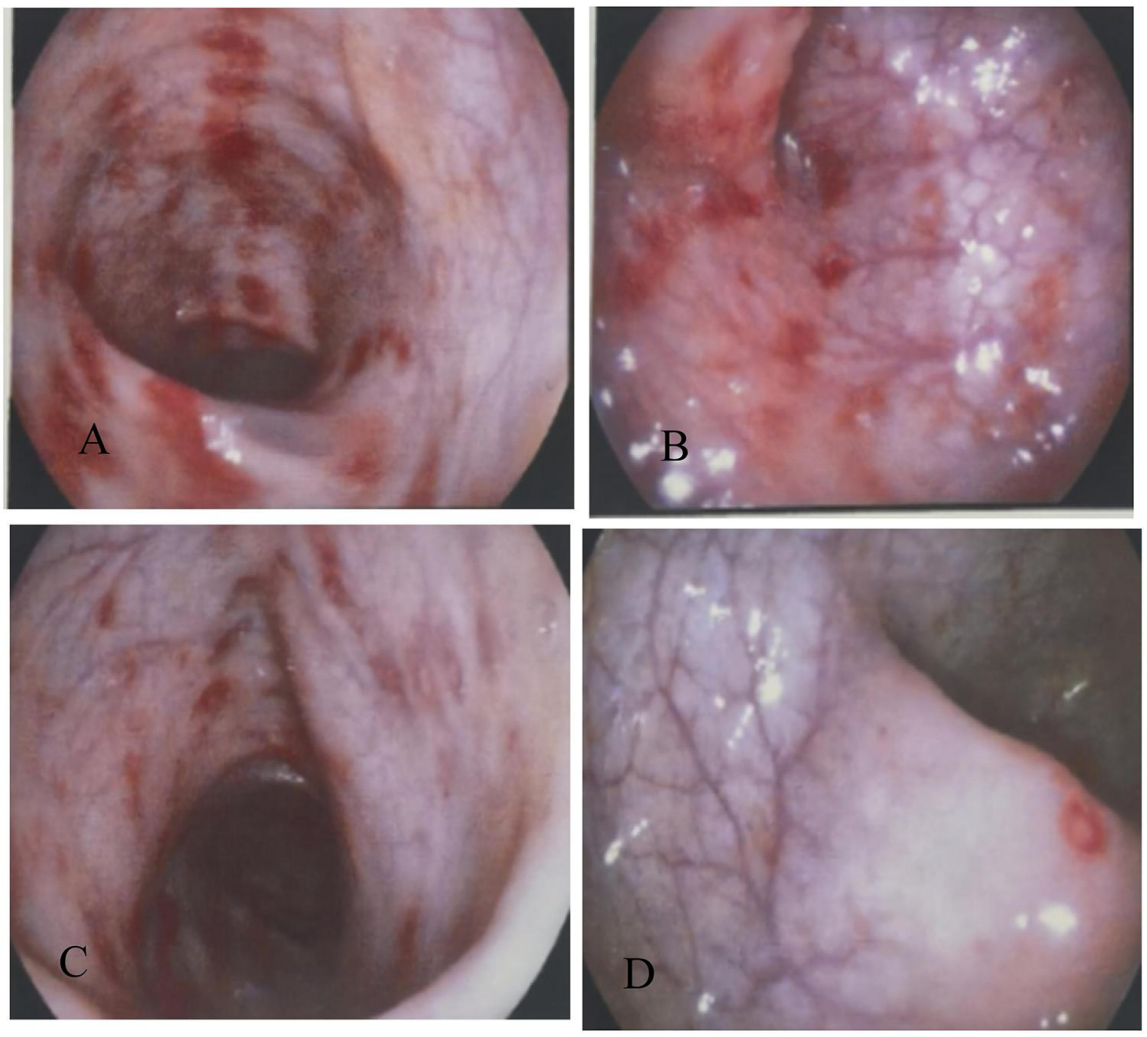
The mucosa of the ascending colon is smooth. Scattered patchy congestion and erosion can be seen in the distal transverse colon **(A)**, descending colon **(B)**, sigmoid colon **(C)** and rectal mucosa **(D)**, which are prone to bleeding upon touch, especially in the descending colon.

**Figure 3:**
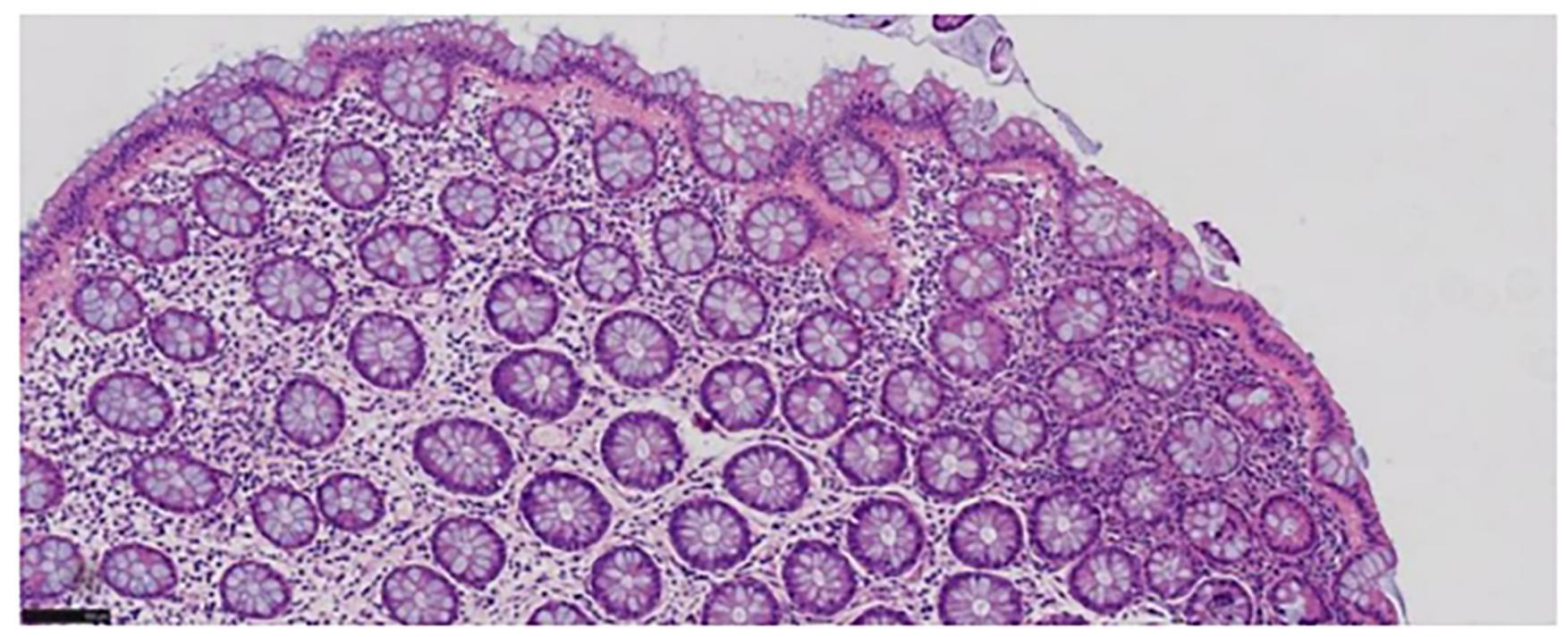
Chronic mucosal inflammation with focal lymphoid tissue hyperplasia. (Magnification 20 × 20 staining: HE).

Laboratory evaluation revealed leukocytosis (WBC 12.31 × 10⁹/L), a positive fecal occult blood test, and markedly elevated antistreptolysin O (ASO) titers (453.00 IU/mL). Blood cultures, stool bacterial and parasitic studies, serum electrolytes, liver and renal function tests, coagulation profile, and autoimmune serologies—including antinuclear antibody (ANA), anti–Saccharomyces cerevisiae antibody (ASCA), and perinuclear antineutrophil cytoplasmic antibody (p-ANCA)—were all within normal limits. Abdominal ultrasound demonstrated no sonographic features of colonic ischemia, including wall thickening, Doppler flow reduction, or intramural gas. Electrocardiography showed sinus arrhythmia—a common, benign rhythm variant in children. Contrast-enhanced abdominal CT revealed mild right pelvic calyceal dilation, multiple small (≤5 mm) mesenteric lymph nodes in the ileocecal region, and no free intraperitoneal fluid or pneumoperitoneum (Figure 1). Colonoscopy identified segmental, patchy mucosal hyperemia and superficial erosions extending from the distal transverse colon to the rectum, with maximal involvement in the descending colon (Figure 2). Histopathological examination demonstrated chronic active colitis characterized by lamina propria lymphoplasmacytosis, crypt architectural distortion, and focal lymphoid aggregates—without evidence of granulomas, vasculitis, or ischemic necrosis (Figure 3).

Following admission, the child remained hemodynamically stable with normal vital signs. Management included bowel rest with clear liquids, intravenous ethamsylate (5 mg/kg/day) for adjunctive hemostasis, intravenous cimetidine (5 mg/kg/day) for gastric mucosal protection. Stool microscopy revealed leukocytes, prompting empiric broad-spectrum antimicrobial therapy with intravenous cefoperazone–sulbactam (100 mg/kg/day) to cover potential enteric pathogens and mitigate bacterial translocation risk. By hospital day 3, abdominal pain and gross hematochezia had resolved, and clinical improvement was sustained. The morning after colonoscopy, the child passed reddish-brown blood clots—consistent with post-biopsy bleeding. A repeat stool analysis showed resolution of fecal leukocytes and erythrocytes; however, fecal occult blood testing remained positive, reflecting residual luminal blood from recent mucosal trauma.

After 7 days of supportive management, the child demonstrated marked clinical improvement—resolution of abdominal pain, cessation of gross hematochezia, and normalization of appetite and activity level. Given the patient's young age, rapid clinical recovery, and the invasive nature and associated risks of diagnostic angiography, the family declined this procedure after thorough shared decision-making. The child was therefore discharged in stable condition.

Post-discharge, oral therapy included Danshen tablets (1 tablet three times daily) to support microcirculatory perfusion, and Bifidobacterium triple viable capsules (1 capsule three times daily) to promote intestinal microbiota balance and mucosal barrier integrity. At 1-month follow-up, the child remained asymptomatic with no recurrence of hematochezia or abdominal symptoms.

## 3 Discussion

IC, an acute or chronic disorder causing colonic mucosal injury due to compromised mesenteric perfusion, is rare in children (2%–5% of all cases) (1). Pediatric cases have different etiologies from adults, mainly involving infectious triggers, vascular anomalies, systemic hypoperfusion, and microcirculatory dysfunction. This report explores IC in children, covering its etiopathogenesis, diagnosis, management, and implications.

### 3.1 Etiology

IC's etiology includes iatrogenic and non—iatrogenic factors. Non—iatrogenic factors range from systemic cardiovascular and metabolic disorders to hemodynamic disturbances and structural vascular abnormalities (8, 9). Iatrogenic factors involve surgical, endovascular, and pharmacologic agents (3, 7). Influenza virus, Hepatitis B virus, novel coronavirus, and *Mycoplasma* infections complicated by IC are thought to be associated with a hypercoagulable state induced by vascular endothelial injury (10–12). In this case, a recent *Streptococcus pyogenes* (group A streptococcus, GAS) infection was suspected to contribute to IC, though no direct link exists. Pediatric-specific risk factors warranting heightened clinical vigilance include congenital vascular anomalies, severe dehydration, acquired or inherited hypercoagulable states, and post-infectious hypoperfusion—each of which should be systematically evaluated during diagnostic workup (10).

The diagnosis was based on clinical, endoscopic, and histopathologic findings, but without mesenteric angiography (gold standard) due to the child's improvement and family's refusal. A structured follow-up plan has been implemented: repeat colonoscopy is scheduled at 6–8 weeks to assess mucosal healing, and CT or MR angiography will be pursued if recurrent symptoms, persistent endoscopic abnormalities, or new clinical concerns arise—enabling noninvasive reassessment of mesenteric vasculature when clinically indicated.

### 3.2 Differential diagnosis

Infectious colitis: This covers bacterial, viral, and parasitic enteritis. The child had fever and elevated stool white blood cells. Yet, blood and stool cultures were negative. There were no typical symptoms like vomiting or mucopurulent bloody stools, and anti—infective treatment failed. Thus, simple infectious enteritis was not diagnosed.

Inflammatory Bowel Disease (IBD): It consists of Crohn's disease and ulcerative colitis, typically presenting as chronic, recurrent abdominal pain, diarrhea, and bloody stools, often accompanied by weight loss and growth retardation. The child in this case had a short disease duration of 6 days and no signs of chronic wasting. Colonoscopy showed no typical IBD features, and the pathology did not reveal non—caseating granulomas. Hence, the diagnosis of IBD was not in line.

Juvenile Polyps: These are a common cause of lower gastrointestinal bleeding in children, usually presenting as painless bloody stools with bright—red blood adhering to the feces' surface. The child in this case had bloody stools with abdominal pain, and no polypoid lesions were seen during colonoscopy. Therefore, this can be ruled out.

Meckel's Diverticulum: It may present as painless massive bloody stools and is frequently associated with diverticulitis or ulcers. The child's lower abdominal CT did not show ectopic gastric mucosa or a diverticulum structure. Although the clinical manifestations were atypical, the possibility of an atypical Meckel's diverticulum should be noted. Further exclusion requires radionuclide scanning or laparoscopic exploration.

Vasculitis—related Enteropathy: Conditions like Henoch—Schönlein purpura, systemic lupus erythematosus, etc., can affect the intestine, causing abdominal pain and bloody stools. The child in this case had no extrarenal manifestations of vasculitis, such as rash, joint swelling and pain, or kidney damage. Complement and autoantibody tests were normal, not supporting the diagnosis of vasculitis.

### 3.3 Diagnostic challenges

IC in children has atypical manifestations. Most cases present with sudden abdominal pain and bloody stools, often with fever and diarrhea. Elevated white blood cells, neutrophils, D—dimer, creatine kinase, along with decreased hemoglobin, metabolic acidosis, and positive fecal occult blood can be diagnostic references (13, 14). Intestinal fatty acid—binding protein (I—FABP) and D—lactate, rapidly released in early acute intestinal ischemia, serve as early diagnostic markers (14).

Imaging examinations:

Abdominal X—ray: May show the “finger—pressure sign” from intestinal wall mucosal edema, indicating lumen narrowing, gas accumulation, colonic band disappearance, and wall thickening. Severe cases may have linear gas in the colonic wall and pneumoperitoneum. Barium examination is not recommended due to ischemia—exacerbating and perforation—inducing risks (15).

Abdominal Computed Tomography (CT): Can reveal wall thickening, intestinal dilation, abdominal effusion, free gas, and obstruction. ⁶⁸Ga-Fibroblast Activation Protein Inhibitor (⁶⁸Ga-FAPI) Positron Emission Tomography/Computed Tomography (PET/CT) can specifically visualize the fibrotic process IC colitis and provide more in—depth pathophysiological insights (16).

Colonoscopy and Pathological Biopsy: Typical endoscopic signs of IC are mucosal congestion, edema, erosion, and ulcers. Lesions are regionally distributed with a clear normal—diseased boundary, and may have submucosal edema or purplish—blue nodules. Pathologically, chronic mucosal inflammation and lymphoid tissue hyperplasia are seen. In this case, endoscopic and pathological results confirmed the diagnosis.

Angiography: As an invasive exam, its clinical use is limited. In this case, the child's rapid improvement led to its postponement.

### 3.4 Management strategies

Rapid and accurate diagnosis of ischemic colitis is crucial for improving patient prognosis and enhancing quality of life. Once diagnosed, proactive measures should be taken to eliminate causative factors, including dietary modifications and gastrointestinal decompression to reduce intestinal oxygen consumption. Timely hemostasis, fluid and electrolyte replenishment, judicious use of broad-spectrum antibiotics, and administration of intestinal mucosal repair agents are essential. When appropriate, vasodilation therapy may be employed to enhance blood circulation and facilitate intestinal recovery. Danshen (Salvia miltiorrhiza) is a traditional Chinese medicine whose active constituents include tanshinones and salvianolic acids. Known pharmacological actions include anti-inflammatory, antioxidant, and microcirculatory improvement effects via inhibition of platelet aggregation and protection of vascular endothelial function (17). However, high-quality evidence for its use in pediatric IC is lacking; its administration in this case was based on adult extrapolation and requires validation in future studies. Probiotics contribute to intestinal mucosal protection and reduce recurrence risk (18). The recurrence rate of ischemic colitis increases over time, necessitating long-term follow-up for this case to monitor signs of recurrence and assess the recovery of intestinal function (6, 7).

The colon possesses a rich vascular supply, with the sigmoid colon, descending colon, and splenic flexure being particularly susceptible to ischemic injury. In cases of diminished systemic perfusion, the right colon is more frequently affected, and the condition tends to be more critical (10, 19). Pediatric IC generally carries a favorable prognosis; however, mortality rates can reach 37%–47% when severe complications such as intestinal obstruction or perforation occur (1). Patients presenting with shock, perforation, or peritonitis may require exploratory laparotomy (20).

## 4 Conclusion

IC is a rare yet clinically significant cause of lower gastrointestinal bleeding in preschool-aged children, frequently presenting with atypical symptoms that increase the risk of misdiagnosis and diagnostic delay. Early colonoscopy—performed within 24–48 h of symptom onset—is the diagnostic cornerstone for children with unexplained hematochezia refractory to standard supportive care. The majority of affected children achieve full clinical and endoscopic recovery with conservative management, yet structured long-term follow-up is essential to detect late complications or recurrence. Heightened clinical awareness, timely endoscopic evaluation, and systematic exclusion of mimicking conditions are critical to minimizing diagnostic delay and optimizing outcomes. The definitive etiology of IC in this case remains undetermined; the GAS-associated hypercoagulability hypothesis, though plausible, requires further investigation in prospective pediatric cohorts.

## Data Availability

The original contributions presented in the study are included in the article/supplementary material, further inquiries can be directed to the corresponding author.
